# Supplementary material for: Flow Cytometry as a New Accessible Method to Evaluate Diagnostic Osmotic Changes in Patients with Red Blood Cell Membrane Defects
Source: Biomedicines. 2024 Jul 19;12(7):1607. doi: 10.3390/biomedicines12071607 (PMC11274888; doi:10.3390/biomedicines12071607)
Supplement: Supplementary file 1 [file biomedicines-12-01607-s001.zip › biomedicines-2939203-supplementary.pdf]

biomedicines

Supplementary materials

Table S1. Normality and Lognormality Test in healthy controls.

	Adults (n=38)			Children (n=16)		
mOsm/Kg	P value	Passed normality test	P value summary	P value	Passed normality test	P value summary
112	0,028	No	*	>0,100	Yes	ns
123	>0,100	Yes	ns	>0,100	Yes	ns
136	>0,100	Yes	ns	>0,100	Yes	ns
176	0,0327	No	*	>0,100	Yes	ns
205	>0,100	Yes	ns	0,0238	No	*
246	0,0276	No	*	0,0141	No	*
308	>0,100	Yes	ns	>0,100	Yes	ns
349	>0,100	Yes	ns	>0,100	Yes	ns
415	0,0122	No	*	0,0077	No	*
458	>0,100	Yes	ns	0,0786	Yes	ns
473	>0,100	Yes	ns	>0,100	Yes	ns
578	>0,100	Yes	ns	>0,100	Yes	ns
585	>0,100	Yes	ns	>0,100	Yes	ns
592	>0,100	Yes	ns	>0,100	Yes	ns

ns; non-significant; * ; significant

Table S2. Normality and Lognormality Test in HS patients

	Adults (n=5)			Children (n=9)		
mOsm/Kg	P value	Passed normality test	P value summary	P value	Passed normality test	P value summary
112	>0,100	Yes	ns	>0,100	Yes	ns
123	>0,100	Yes	ns	>0,100	Yes	ns
136	>0,100	Yes	ns	>0,100	Yes	ns
176	0,0112	No	*	0,0469	No	*
205	0,0026	No	**	>0,100	Yes	ns
246	>0,100	Yes	ns	>0,100	Yes	ns
308	>0,100	Yes	ns	>0,100	Yes	ns
349	>0,100	Yes	ns	>0,100	Yes	ns
415	>0,100	Yes	ns	>0,100	Yes	ns
458	>0,100	Yes	ns	>0,100	Yes	ns
473	>0,100	Yes	ns	>0,100	Yes	ns
578	>0,100	Yes	ns	>0,100	Yes	ns
585	0,0403	No	*	>0,100	Yes	ns
592	>0,100	Yes	ns	>0,100	Yes	ns

ns; non-significant, *; significant
